# Pilot-job provisioning on grid resources: Collecting analysis and performance evaluation data

**DOI:** 10.1016/j.dib.2022.108104

**Published:** 2022-03-26

**Authors:** Alexandre F. Boyer, Christophe Haen, Federico Stagni, David R.C. Hill

**Affiliations:** a European Organization for Nuclear Research, Meyrin, Switzerland; b Université Clermont Auvergne, Clermont Auvergne INP, CNRS, Mines Saint-Etienne, LIMOS, 63000 Clermont-Ferrand, France

**Keywords:** Grid computing, Pilot-job, DIRAC site director, LHCb

## Abstract

To take advantage of the computing power offered by grid and opportunistic resources, the CERN Large Hadron Collider (LHC) experiments have adopted the Pilot-Job paradigm. In this work, we study the DIRAC Site Director, one of the existing Pilot-Job provisioning solutions, mainly developed and used by the beauty experiment (LHCb). The purpose is to improve the Pilot-Job submission rates and the throughput of the jobs on grid resources. To analyze the DIRAC Site Director mechanisms and assess our contributions, we collected data over 12 months from the LHCbDIRAC instance. We extracted data from the DIRAC databases and the logs. Data include (i) evolution of the number of Pilot-Jobs/jobs over time; (ii) slots available in grid Sites; (iii) number of jobs processed per Pilot-Job.

## Specifications Table

**Table:** 

Subject	Software Engineering
Specific subject area	Analysis and evaluation of the Pilot-Job provisioning on grid resources
Type of data	Text, Comma Separated Files (CSV), JavaScript Object Notation (JSON), Figures
How data were acquired	A Local Resource Management System (LRMS) of a grid site provides a state of the computing resources and their usage. Computing Elements (CEs) aggregate data from one or multiple LRMS and record information about jobs and Pilot-Jobs. They transfer data between grid sites and Workload Management System (WMS). A Site Director generates Pilot-Jobs, stores related data in a *PilotAgentsDB* database and an accounting service (DIRAC Accounting service). It supplements Pilot-Jobs data with information coming from CEs and produces logs containing details about the operations performed.
Data format	Raw, Filtered, Analyzed
Description of data collection	Data collected provided insights about the limits of the Site Director and the impact of our contributions to improve the throughput of the jobs on grid resources. Data collection was performed for 12 months and targeted a specific group of Site Directors. It was split into three different phases: (i) the first phase (4 months) represents the original state of the group; (ii) the second phase (4 months) is related to a contribution introduced in production; (iii) in the same way, the last phase (4 months) corresponds to another contribution. The evolution of the number of jobs processed in parallel and the number of Pilot-Jobs submitted per hour was collected at the end of the analysis from the DIRAC web interface, which interacts with the Accounting service. Further details about the operations of the Site Directors and the activities of the Pilot-Job were extracted, from the log files and from client interfaces interacting with the *PilotAgentsDB* database and the CEs, for a short period (a few hours), multiple times per phase.
Data source location	Institution: European Organization for Nuclear Research (CERN)
	City: Meyrin
	Country: Switzerland
	Latitude and longitude: 46.2338702,6.0469869
Data accessibility	Repository name: DIRAC Site Director: Analysis and Performance Evaluation
	Data identification number: 10.17632/6r388827fz.2
	Direct URL to data: https://data.mendeley.com/datasets/6r388827fz/2
Related research article	Alexandre F. Boyer, Christophe Haen, Federico Stagni, David R.C. Hill, DIRAC Site Director: Improving Pilot-Job provisioning on grid resources, Future Generation Computer Systems, Volume 133, 2022, Pages 23-38, ISSN 0167-739X, https://doi.org/10.1016/j.future.2022.03.002.

## Value of the Data

• This dataset provides metrics and directions to analyze and assess the efficiency of a Pilot-Job provisioning system on grid resources.
• DIRAC [1], [2] is an open-source interware used by various experiments such as LHCb, Belle II [3] and CTA [4]; in different contexts: WLCG, EGI. DIRAC administrators could directly reuse this work. This dataset could also provide insights and guidance to any virtual organization applying the Pilot-Job paradigm on grid resources.
• These data might be used to analyze the limits of a Pilot-Job provisioning tool, to assess the scaling capabilities of performance improvement contributions.
• These data could potentially help virtual organizations to better understand the functioning of grid computing resources, and thus to better exploit them.

## 1 Data Description

The dataset is split into two parts:

• Resources: raw data coming from LHCbDIRAC, divided into subsections according to the nature of the data. Each subsection contains data along with a short markdown description (README.md) and a set of Python programs used to extract and filter data (tools). They are proposed and designed in order to facilitate the reproducibility of the experiments.
• Results: processed data including figures and tables. They are produced by a Jupyter notebook program (DIRACSiteDirector.ipynb) exploiting raw data.

### 1.1 Raw data

Grid computing resources are heterogeneous and volatile. They are owned by many different institutes across the world and shared by several virtual organizations. Thus, an exhaustive analysis of a Pilot-Job provisioning system requires data from many remote sources. In the Resources directory, information is sorted into subsections according to its source and nature:

• configInfo: gives information about associations between Site Directors, sites and CEs: (i) cesPerSite.json is a dictionary where the Site Director name is the key and names of the CEs bound to it is the value. The CE identifier contains the grid name, the site name and the CE name such as $grid_{ID}.site_{ID}.ce_{ID}$. (ii) siteDirectortypeCE.csv is another dictionary bounding a type of CE to a Site Director. Indeed, in LHCbDIRAC, administrators chose to associate a Site Director to a single type of CE. Information comes from the DIRAC configuration service relying on BDII [5], a database centralizing data from grid sites and CEs.
• jobsPerPilot: provides a table representing the average number of jobs fetched and processed per Pilot-Job, grouped by CE, in a month (jobsPerPilot.csv). Each line corresponds to a day, each column to a CE, and the values to the average number of jobs processed per Pilot-Job. Data come from the Accounting service and were extracted from the DIRAC web interface.
• pilot-jobsSubmitted: represents the evolution of (i) the number of Pilot-Jobs submitted per hour (pilotsSubmitted); (ii) the number of jobs processed in parallel (jobsProcessed). pilotsSubmitted contains two files: failed-010120-190121.csv the evolution of the failed submission of pilots and success-010120-190121.csv the progression of the successfully submitted pilots. In both files, each line represents a week, each column a Site Director and values the average number of pilots submitted by a given Site Director for a given week. jobsProcessed has one file (010120-190121.csv): each line corresponds to a week, each column to a grid site, and the values to the average number of jobs processed by a given site for a given week. Files were downloaded from the Accounting service through the DIRAC web interface.
• submission-matchingTime: is composed of two tables: (i) pilotDuration.csv and jobDuration.csv. pilotDuration.csv represents the time a Pilot-Job spends from its submission to its execution on a computing resource. Each line represents a Pilot-Job. Columns 1, 4 and 5 provide information about sites and CEs involved and columns 2 and 3 are the installation date and the submission date, respectively. jobDuration.csv is relatively similar but contains fewer details. In this file, each line describes a job as two dates: the moment when the job arrives in DIRAC, the moment when the job is fetched by a Pilot-Job running in a grid site. To produce the files, one has to (i) get job and Pilot-Job identifiers from the DIRAC web interface; (ii) call DIRAC client interfaces interrogating the DIRAC databases to get further details about the chosen entities.
• pilotsActivities: contains information about the statuses of the Pilot-Jobs under the form of JSON files. The directory contains several files named result_sorted_<date>, where *date* corresponds to the moment where data were extracted. Each file contains the number of Pilot-Jobs grouped by status and by date. Values were extracted every 5 min for 12 h using a DIRAC client interface interacting with the *PilotAgentsDB* database.
• individualEvaluation: used to assess individual contributions brought to the Pilot-Job provisioning tool. arcEvalOutput.csv is the result of 3 executions of a script that compared two methods to get the status of a variable number of Pilot-Jobs on 3 ARC CEs. creamEvalOutput.csv was generated by a script, executed 5 times, comparing two methods to renew the proxy of 2 CREAM CEs [6]. parallelEvalOutput.csv was built by a program comparing the monitoring operations of a Site Director interacting with a variable number of CEs - from 1 to 5 - with (i) one thread and (ii) multiple threads.
• logs: embeds several directories named <date> corresponding to the date of extractions of the underlying log files. Each directory contains log files bound to Site Directors. Logs have information of interests that have to be extracted, such as the number of slots available in the grid sites, the number of pilots submitted and the duration of the operations.

### 1.2 Processed data

Once collected from different sources, data have to be combined and easily readable to provide insights to the developers. DIRACSiteDirector.ipynb was used to process raw data from Resources in order to generate figures and tables in Results:

• jobsPerPilot.pdf: generated from Resources/jobsPerPilot under the form of a heat map.
• submission-matching.pdf: box plot combining data from Resources/submission-matchingTime. It compares the duration from the pilot generation to the pilot installation on a worker node to the duration from the job arrival to the job matching.
• pilotActivities.pdf: combines information coming from Resources/pilotsActivities and Resources/logs to highlight the variations Pilot-Jobs in certain sites.
• SDsMonitoring.pdf: compares duration of the operations using data from the logs (Resources/logs).
• individualEval<contribution>.pdf: highlight the results of a given contribution.
• cpuTimeUsedPerSecond.pdf and pilotsSubmittedPerHour.pdf: represents the evolution of the number of jobs processed in parallel and the number of pilots submitted, respectively, in bar plots. They both use data from Resources/pilot-jobsSubmitted
• runningPilots.pdf and scheduledPilots.pdf: box plots using Pilot-Jobs activities information located in Resources/pilotsActivities. It represents the evolution of the number of running and waiting pilots through different phases.
• monitoringNumberPilotsSubmitted.pdf: plot describing the evolution of the monitoring time and the number of Pilot-Jobs submitted per hour through different phases. Data comes from logs and raw data from various subsections of Resources and involves 13 Site Directors.
• errorsPerSD.tex: is a table containing the number of failed submissions observed in Site Directors grouped by phase. Data comes from Resources/pilot-jobsSubmitted.
• numberOfPilotsSubmittedEvolution.pdf: box plot relying on logs showing the evolution of the number of pilots submitted per cycle of Site Director, through different phases.

## 2 Experimental Design, Materials and Methods

### 2.1 Getting data from grid resources

Getting data from a large number of heterogeneous and remote computing resources require centralization mechanisms at some point:

• Administrators can install BDII agents on grid sites, which are able to collect LRMS configuration data (Fig. 1.1.1 and 1.1.2). These data are centralized and can directly be used by WMS.

**Fig. 1:**
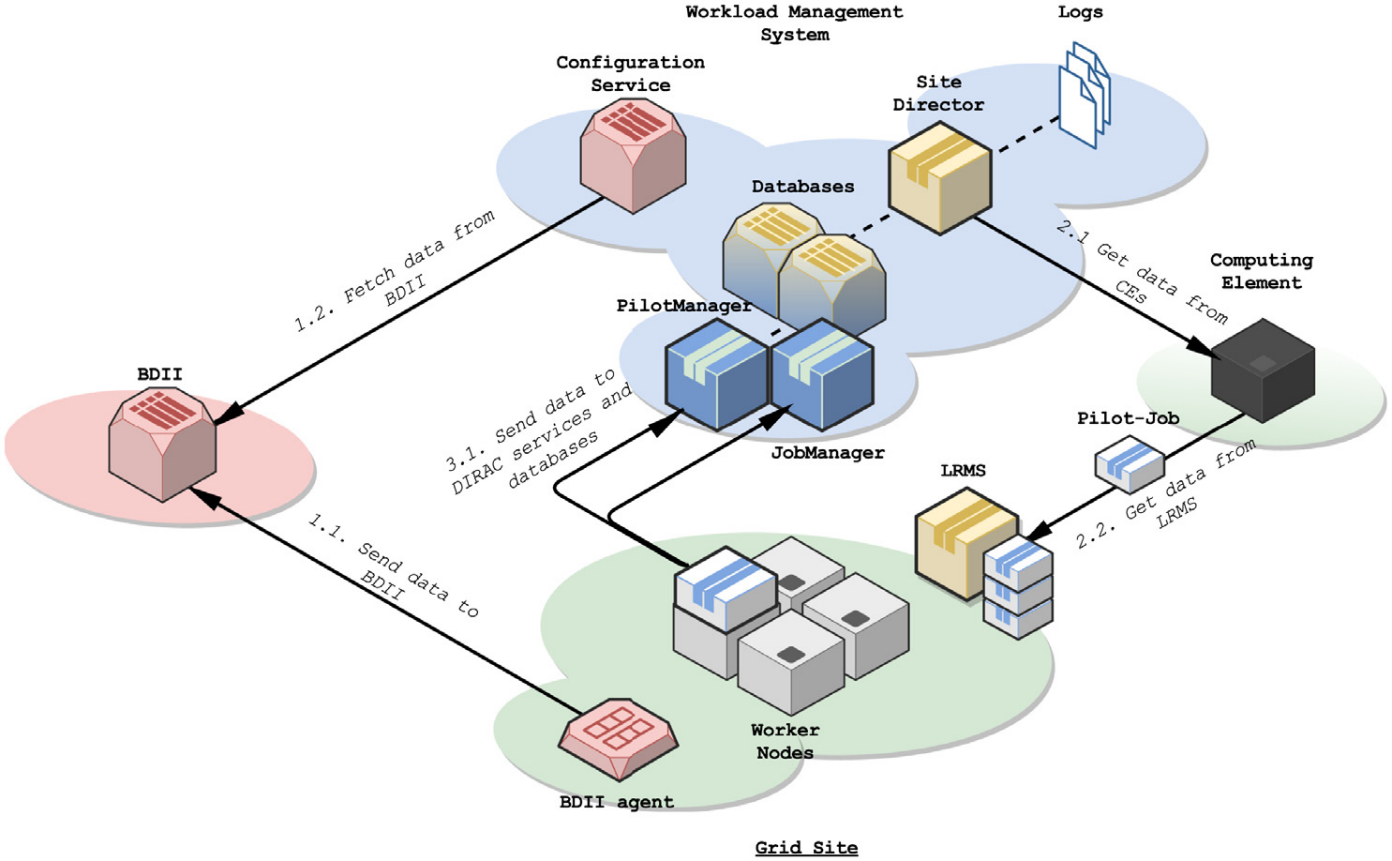
Interactions between grid components to centralize data.
• LRMS orchestrate computing resources of grid sites and gather data about the state and the use of the resources to schedule jobs. To ease the interactions with a large number of grid sites with various types and versions of LRMS, WMS deal with entry points called CEs. CEs receive jobs and Pilot-Jobs from WMS and transfer them to a LRMS of a grid site. They generally embed a service to record and send information about the Pilot-Jobs and their status. A Pilot-Job provisioning tool - such as the DIRAC Site Director - generally acts as a centralization point: (i) it generates and submits Pilot-Jobs to different CEs bound to several grid sites; (ii) it gets information about the submitted Pilot-Jobs and records information in one or more databases (Fig. 1.2.1 and 1.2.2).
• Pilot-Jobs, once installed, communicate information about worker nodes to the WMS services. They are also stored in one or more databases (Fig. 1.3.1).

### 2.2 Collecting pilot-jobs provisioning related data

The DIRAC interware provides different means to interact with WMS-related data:

• A web interface: an administrator can generate plots and CSV files about Pilot-Jobs and jobs, for a given period. The web interface is linked to (i) the Accounting service aggregating data from many DIRAC services; (ii) a *JobManager* service to interact with the *JobDB* database; (iii) a *PilotManager* service to get information from the *PilotAgentsDB*.
• Command-line interfaces: in the same way, an administrator can use command-line interfaces from a terminal to interrogate DIRAC databases.
• Logs: DIRAC services produces logs that are stored in files. Access to the DIRAC server is generally required.

We collected data about Pilot-Jobs and jobs related to the LHCb experiment offline activities on WLCG for 12 months split into 3 phases. The experiment involved a group of 13 Site Directors supplying 65 sites with Pilot-Jobs. They were interacting with different types of CEs and LRMS. During the first phase (4 months), we analyzed the Site Directors to find their limits. The second phase (4 months) started after we introduced changes: we decreased the number of communications and their duration with CEs. Finally, the third phase (4 months) began after we configured Site Directors to submit Pilot-Jobs more frequently.

The process was similar for the 3 phases. We extracted the logs of the Site Director that contained 2 to 3 days of information, and we accessed database information, multiple times for short periods, to produce average results (Fig. 2.1.1). Getting average values limits the bias that could be introduced if grid sites are in maintenance for instance. We also got CSV files from the web application to perform an analysis in the long term and at a large scale (Fig. 2.1.2).

**Fig. 2:**
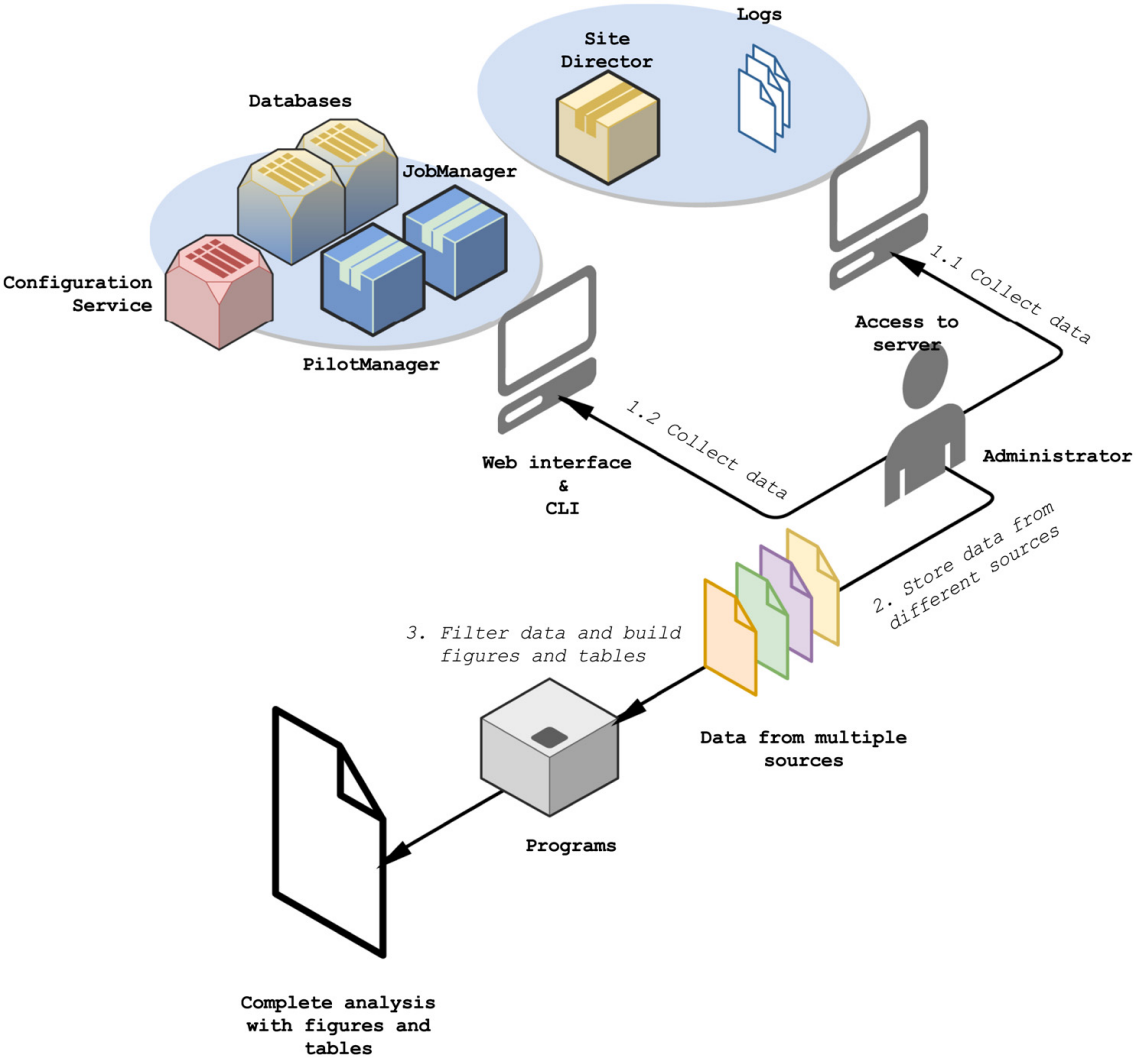
Workflow: collecting and processing WMS data.

### 2.3 Extracting knowledge from raw data

We ended up with data from different sources, and we wanted to combine them to build knowledge that would help to improve the Pilot-Job provisioning tool (Fig. 2.2). First, we designed programs to filter information from raw and processed data: site and CE names were replaced by identifiers. Then, we created a Jupyter notebook to import log, CSV and JSON files - filtered - and we built programs to combine data and generate figures and tables 2.3).

## Ethics Statement

The authors state that they have no ethical issue in this work since data were collected in a control experiment using open source software, manuals, and publicly available referenced articles.

## CRediT authorship contribution statement

**Alexandre F. Boyer:** Conceptualization, Methodology, Software, Formal analysis, Investigation, Data curation, Writing – original draft. **Christophe Haen:** Conceptualization, Supervision, Validation. **Federico Stagni:** Project administration, Resources. **David R.C. Hill:** Supervision, Writing – review & editing, Validation.

## Declaration of Competing Interest

The authors declare that they have no known competing financial interests or personal relationships which have, or could be perceived to have, influenced the work reported in this article.

## Data Availability

Pilot-Job provisioning on Grid Resources: Collecting Analysis and Performance Evaluation Data (Original data) (Mendeley Data).
